# CO_2_ induced seawater acidification impacts survival and development of European eel embryos

**DOI:** 10.1371/journal.pone.0267228

**Published:** 2022-04-18

**Authors:** Daniela E. Sganga, Flemming T. Dahlke, Sune R. Sørensen, Ian A. E. Butts, Jonna Tomkiewicz, David Mazurais, Arianna Servili, Francesca Bertolini, Sebastian N. Politis

**Affiliations:** 1 National Institute of Aquatic Resources, Technical University of Denmark, Kgs. Lyngby, Denmark; 2 Thünen Institute of Fisheries Ecology, Bremerhaven, Germany; 3 Billund Aquaculture, Billund, Denmark; 4 School of Fisheries, Aquaculture and Aquatic Sciences, Auburn University, Auburn, Alabama, United States of America; 5 CNRS, IRD, LEMAR, Ifremer, Université de Brest, Plouzané, France; University of Connecticut, UNITED STATES

## Abstract

Fish embryos may be vulnerable to seawater acidification resulting from anthropogenic carbon dioxide (CO_2_) emissions or from excessive biological CO_2_ production in aquaculture systems. This study investigated CO_2_ effects on embryos of the European eel (*Anguilla anguilla*), a catadromous fish that is considered at risk from climate change and that is targeted for hatchery production to sustain aquaculture of the species. Eel embryos were reared in three independent recirculation systems with different pH/CO_2_ levels representing “control” (pH 8.1, 300 μatm CO_2_), end-of-century climate change (“intermediate”, pH 7.6, 900 μatm CO_2_) and “extreme” aquaculture conditions (pH 7.1, 3000 μatm CO_2_). Sensitivity analyses were conducted at 4, 24, and 48 hours post-fertilization (hpf) by focusing on development, survival, and expression of genes related to acute stress response (*crhr1*, *crfr2*), stress/repair response (*hsp70*, *hsp90*), water and solute transport (*aqp1*, *aqp3*), acid-base regulation (*nkcc1a*, *ncc*, *car15*), and inhibitory neurotransmission (*GABAAα6b*, *Gabra1*). Results revealed that embryos developing at intermediate pH showed similar survival rates to the control, but egg swelling was impaired, resulting in a reduction in egg size with decreasing pH. Embryos exposed to extreme pH had 0.6-fold decrease in survival at 24 hpf and a 0.3-fold change at 48 compared to the control. These observed effects of acidification were not reflected by changes in expression of any of the here studied genes. On the contrary, differential expression was observed along embryonic development independent of treatment, indicating that the underlying regulating systems are under development and that embryos are limited in their ability to regulate molecular responses to acidification. In conclusion, exposure to predicted end-of-century ocean pCO_2_ conditions may affect normal development of this species in nature during sensitive early life history stages with limited physiological response capacities, while extreme acidification will negatively influence embryonic survival and development under hatchery conditions.

## 1. Introduction

Ocean acidification (OA) results from the uptake of increasing anthropogenic levels of carbon dioxide (CO_2_), which dissolves in water causing a decline in pH [1]. Seawater pCO_2_ in the ocean increases along with atmospheric CO_2_ levels [2]. Studies have estimated that up to 25% of CO_2_ emissions, derived from human activities, has been taken up by the ocean causing a decline in global mean surface pH of approximately 0.1 units relative to preindustrial values [3]. Exposure to predicted end-of-century conditions (~1,000 μatm) could impact aquatic organisms [4], where growing experimental evidence is suggesting that OA can negatively affect fish early life development, growth [5, 6], reproduction [7], and behaviour [8, 9].

Adult fish usually have relatively efficient mechanisms for acid-base regulation [10]. Disturbance of acid-base balance due to increased seawater CO_2_ concentrations is compensated by excretion of protons (H^+^) via the gills and accumulation of bicarbonate ions (HCO_3_^-^) in body fluids. These processes involve various ion transport proteins, such as Na^+^K^+^2Cl^−^ (nkcc), and Na^+^Cl^−^ (ncc) cotransporters [10, 11]. In addition, CO_2_ diffusion is mediated by members of the aquaporin gene family [12]. At pH levels consistent with OA, the alterations in HCO_3_^−^ and Cl^−^ gradients can also affect the function of the gamma-aminobutyric acid A receptor (GABA_A_), the main neurotransmitter receptor in the brain, leading to behavioural alterations [13]. In particular, upregulation of the GABA_A_ receptor α subunits has been observed after exposure to high CO_2_ [14, 15]. At the same time, high O_2_ consumption, resulting from increased ion exchange across the gill surface and associated ATP demand, can lead to the formation of reactive oxygen species (ROS). This triggers a cellular stress/repair response, in which the expression of molecular chaperones, such as heat shock proteins (*hsp*) are upregulated [16].

Fish embryos and larvae are usually more sensitive to pH changes than adults, as organs (e.g., gills) for pH regulation are not fully developed [11, 17, 18]. Homeostatic capacities may increase with the development of specialized cells and tissues after gastrulation [18]. It is also expected that after this critical period, embryos are increasingly able to respond to acid-base balance challenges through changes in the expression of genes encoding relevant proteins (i.e., developmental plasticity) [18, 19]. However, elevated metabolic costs associated with ion transport mechanisms can lead to reduced growth and other downstream consequences, including neurophysiological impairments [11]. As such, concerns arise regarding the potential effects of OA on early life stages of fish species spawning in the ocean, such as the European eel, *Anguilla anguilla*.

The European eel life cycle involves continental juvenile stages followed by oceanic reproductive and larval stages. Spawning takes place in the Sargasso Sea and leptocephalus larvae are transported to the north-eastern Atlantic coasts where they metamorphose to glass eels [20, 21]. Currently, the European eel stock is listed as “critically endangered” on the IUCN Red List [22] and considered outside of safe biological limits, mainly due to anthropogenic stressors and ongoing climate change [23–27]. Eels are also high-value species in aquaculture, where they are farmed in resource-efficient recirculating aquaculture systems (RAS) [28]. However, production is still based on a supply of juveniles (glass eels) caught in nature. Thus, dedicated efforts are made towards closing the European eel life cycle in captivity [29, 30]. This includes identification of optimal environmental conditions for embryonic and larval rearing, such as light [31, 32], salinity [33–35], and temperature [36–38], as knowledge about their natural reproduction, spawning habitats, and offspring ecophysiology is scarce.

The effects of pH fluctuations on European eel are largely unknown. Yellow eels can tolerate elevated CO_2_ levels in the blood (hypercapnia) during short-term exposure to acidification, showing no effect on aerobic metabolism and plasma levels of catecholamine and cortisol, which typically increase in response to environmental stress [39, 40]. Likewise, glass eels’ survival rates were not affected by long-term exposure to a pH of 7.6 [41], although a decrease in heat shock response and in the antioxidant enzymatic machinery was observed [42]. The effect of decreased pH on eel early life stages has not been investigated. In this regard, evaluating the sensitivity of eel offspring to fluctuations in pCO_2_/pH, and identifying the developmental timing and functionality of related regulation pathways may help predicting the effects of ocean acidification on reproductive success and recruitment. Concurrently, such information can be included in the optimization of water quality (CO_2_/pH) management in eel hatcheries that use RAS technology. In RAS, high CO_2_/low pH conditions can result from microbial activity in biofilters, with extreme values of up to pH 5 observed under intensive rearing conditions [28].

This study experimentally assessed the sensitivity of hatchery produced European eel offspring to CO_2_-induced water acidification (pH_NBS_ 7.1, 7.6, and 8.1) at 4, 24, and 48 hours post-fertilization (hpf) by focusing on development and survival of eel embryos. Furthermore, embryonic changes in expression of genes involved in acute stress responses (*crhr1*, *crfr2*), stress/repair responses (*hsp70*, *hsp90*), water and solute transport (*aqp1*, *aqp3*), acid-base regulation (*nkcc1a*, *ncc*, *car15*), and inhibitory neurotransmission (*GABAAα6b*, *Gabra1*) were explored. We hypothesized that exposure to acidified seawater can result in an upregulation of these genes as part of a compensatory mechanism, restoring acid-base balance. At the same time, we hypothesized that eel embryos can be sensitive to acidification, which would be reflected in an increased mortality at low water pH.

## 2. Materials and methods

### 2.1. Ethics statement

As exclusively embryonic stages of European eel were used, no licence is required according to Danish and European Union regulations.

### 2.2. Experimental design

The experiment was repeated three times with fertilized eggs from different parental crosses (n = 3) and these crosses were treated as biological replicates for all statistical analyses. The embryos of each cross were incubated at optimum temperature (18°C, [37]), salinity (36, [35]), and three pCO_2_/pH conditions: (1) control-pH with pH 8.1 and 300 μatm CO_2_, (2) intermediate-pH with pH 7.6 and 900 μatm, and (3) extreme-pH with pH 7.1 and 3000 μatm (Table 1). The control and intermediate pH treatments correspond respectively to current surface pCO_2_/pH conditions in the putative eel spawning area in the Sargasso Sea [43] and the extent of global ocean acidification projected by 2100 under a high emission scenario [44]. The extreme-pH treatment was selected to reflect conditions that may occur in RAS systems due to high bacterial activity from nitrifying and heterotrophic bacteria in biofilters and bacteria in hatchery water or from fish metabolism when reared at high densities, i.e., CO_2_ production [45]. Treatment effects on eel development were assessed based on embryonic survival at 4, 24, and 48 hpf, and embryonic morphometrics at 4 (i.e., irregular cleavages) and 24 hpf (i.e., chorion diameter, perivitelline space area). In addition, gene expression was assessed on embryos sampled at 24 and 48 hpf (detailed below).

**Table 1:** European eel, *Anguilla anguilla* embryos were reared in a series of closed recirculation aquaculture systems, each with a different pCO_2_/pH treatment.

Parameter	Control-pH	Intermediate-pH	Extreme-pH
Salinity	36.25 ± 0.22	36.28 ± 0.22	36.51 ± 0.42
Temperature (°C)	17.88 ± 0.53	18.03 ± 0.22	18.46 ± 0.20
pH_NBS_	8.08 ± 0.02	7.64 ± 0.07	7.16 ± 0.05
pH_T_	7.94 ± 0.01	7.51 ± 0.05	7.05 ± 0.04
TA (mg CaCO_3_/kg)	128.65 ± 1.37	131.88 ± 1.52	149.58 ± 4.15
pCO_2_ (μatm)	290,04 ± 12,96	892.71 ± 123.71	3122.69 ± 246.13
HCO_3_^−^ (μmol/kg)	1059.36 ± 17.61	1220.37 ± 21.54	1456.56 ± 37.92
CO_3_^2−^ (μmol/kg)	78.74 ± 1.84	34.59 ± 3.70	14.38 ± 1.89
DIC (μmol/kg)	1147.96 ± 16.79	1285.13 ± 22.40	1575.10 ± 36.82

Mean ± SD (of three replicate parental crosses) water quality parameters for each pCO_2_/pH treatment are reported. pH_T_ was calculated from the measured pH_NBS_ values. Partial pressure of CO_2_ (pCO_2_), bicarbonate ion concentration (HCO_3_^−^), carbonate ion concentration (CO_3_^2−^), and dissolved inorganic carbon (DIC) were calculated based on pH_T_, total alkalinity (TA), salinity and temperature [55].

### 2.3. Fertilization

Fertilised eggs of European eel were obtained from the prototype hatchery, EEL-HATCH, managed by the Technical University of Denmark. Gametes originated from wild-caught female broodstock and farmed-raised male broodstock reared at 20°C, applying assisted reproduction protocols for the induction of gametogenesis and final maturation [46–48]. Eggs from each female were fertilized separately by different sperm pools of several males [49] to create 3 parental crosses, using standardised fertilisation procedures [35, 50]. Fertilized eggs were then incubated at a salinity of 36, 18°C, and pH 8.1 for 2 h. Egg density was determined by counting 3 × 0.1 mL subsamples of the floating layer. Only floating viable embryos were used for experimentation.

### 2.4. Embryo incubation

**Fig 1:**
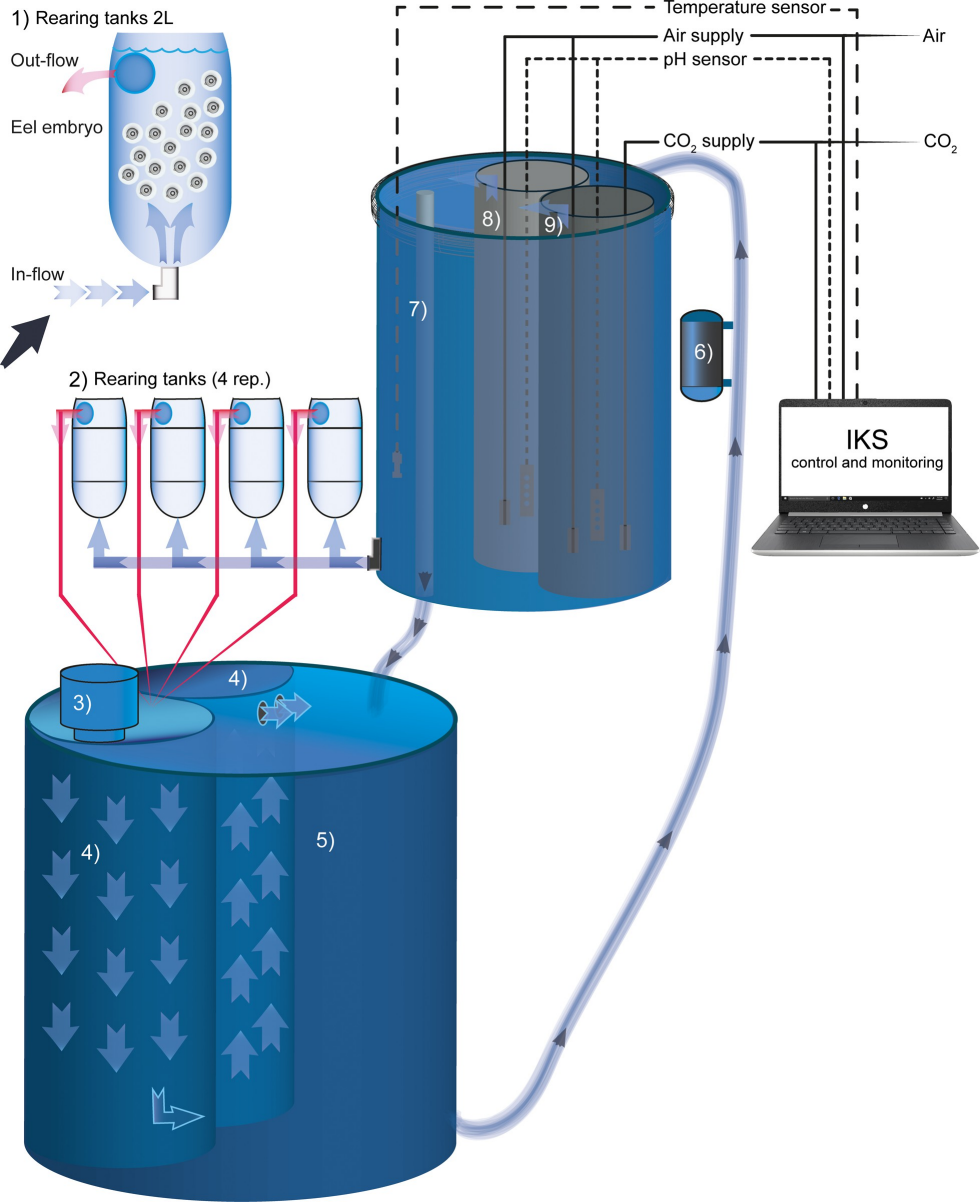
European eel, *Anguilla anguilla* embryos from each treatment were reared in upwelling, 2 L incubators (n = 3) connected to a recirculating aquaculture system (RAS). Each RAS unit consisted of a sump reservoir (bio-filter, protein skimmer and UV sterilizer, 250 L volume) followed by a similar sized header tank, which supplied 4 incubators (3 containing ~150 embryos per 100 mL and a “blank” incubator for water measurements), with a water exchange rate of ~0.3 L per min. A multi-channel feedback system controlled the pCO_2_/pH conditions within the RAS units by measuring pH in the header tank and infusing pure CO_2_ gas into the water to maintain the target pH value at the high and extreme-pCO_2_ treatments. (1) rearing incubator, (2) rearing incubator replicates, (3) protein skimmer, (4) bio-filter, (5) reservoir, (6) UV-lamp, (7) header tank, (8) air supply, pH regulation (9) CO_2_ supply, pH regulation.

Environmental pCO_2_/pH conditions were controlled in three custom designed RAS units (Fig 1), each containing 500 L of filtered and UV-sterilized natural North Sea seawater with adjusted salinity (36; Aquaforest Reef Salt, Brzesko, Poland). Each RAS system consisted of a filtration sump (biofilter, protein skimmer and UV sterilizer, 250 L volume) and a header tank (250 L volume) supplying four upwelling incubators (2 L volume each). Three of the four incubators were stocked with ~150 fertilized eggs (embryos) per 100 mL volume and the water exchange rate was set to ~0.3 L per min. The desired pCO_2_/pH conditions within the RAS units were controlled using a multi-channel feedback system (IKS Aquastar, IKS Computer Systems, Karlsbad, Germany), which continuously measured the seawater pH of the header tank and infused pure CO_2_ gas into the water to maintain the target pH value (intermediate-pH or extreme-pH). To ensure rapid CO_2_ dissolution and minimize pCO_2_/pH fluctuations within the incubators, the water in the header tanks was mixed by injection of compressed air. No CO_2_ was added to the RAS unit used as the control (pH 8.1). Seawater pH and temperature in the header tanks was recorded automatically every 30 min by the IKS system. In addition, manual (daily) measurements of seawater pH (HQ11D Portable pH Meter, Hach, USA) were taken. The electrodes were calibrated weekly to NBS buffers 4.0, 7.0, and 10.0. Water samples were taken at the beginning and end of the experimental period from the blank incubators, for determining total alkalinity (TA) by acid titration [51]. The measured pH_NBS_ was used to calculate pH_T_ based on conversion constants using the AquaEnv package [52] for R [53], as no Tris buffer reference material was available for empirical conversion of pH_NBS_/pH_T_ [54]. It is therefore likely that the calculated pH_T_ values are about 0.1 units higher and the estimated pCO_2_ values correspondingly 10–20% lower than the true values. Other parameters of the carbonate system were calculated from pH_T_, TA, salinity, and temperature using the package seacarb [55] for R. All water quality parameters are summarized in Table 1.

### 2.5. Data collection

#### 2.5.1. Embryonic survival and morphological measurements

**Fig 2:**
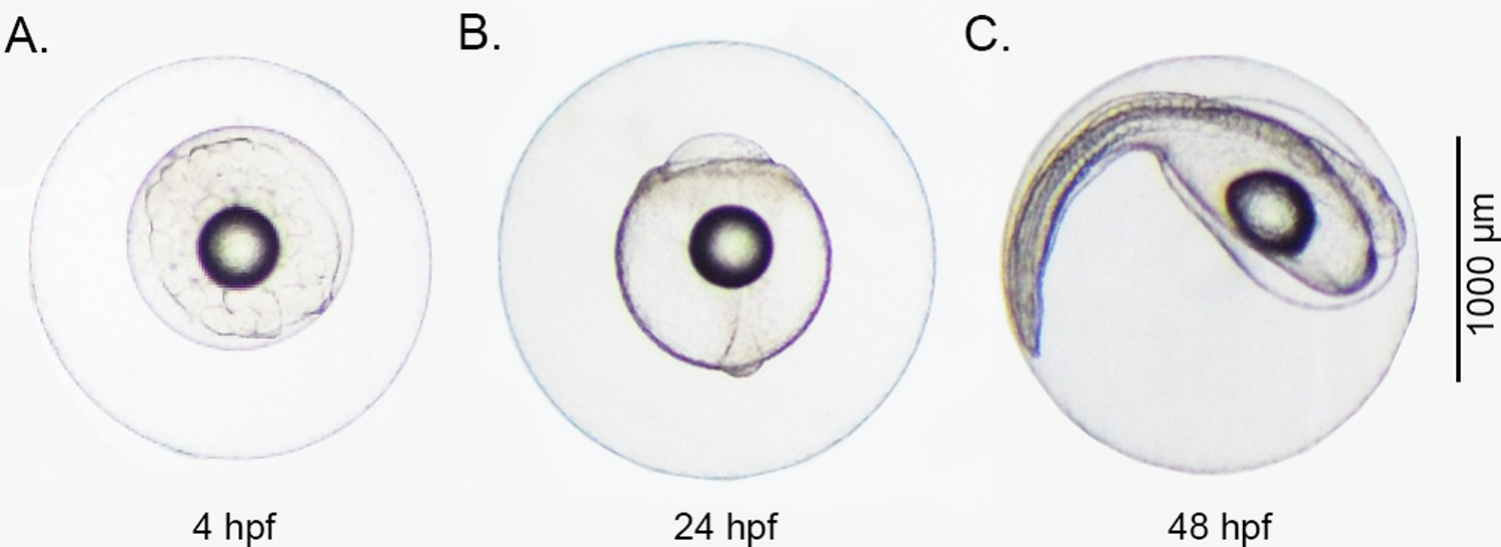
Developing European eel, *Anguilla anguilla* embryos at (A) 4, (B) 24, and (C) 48 hours post-fertilization, incubated at 18°C under “control” hatchery conditions.

Dead embryos were removed from the bottom of the incubator and counted at 4, 24, and 48 hpf for assessment of embryonic survival. Embryos (n = ~20) from the floating layer from each replicate were randomly sampled at 4 hpf (Fig 2A) and imaged using a digital camera (Digital Sight DS-Fi1, Nikon Corporation, Japan) attached to an objective microscope (Eclipse 55i, Nikon Corporation, Japan) and categorized according to irregular cell cleavage patterns following [35]. Subsequent samples were taken at 24 hpf (Fig 2B) and digitally imaged for measuring chorion diameter and perivitelline space area. NIS-Elements D analysis software (version 3.2) was used to analyse the images (Nikon Corporation, Japan).

#### 2.5.2. Gene expression

For molecular analysis, a pool of ~20 embryos from each replicate were randomly sampled at 24 and 48 hpf (Fig 2B and 2C), preserved in RNAlater^®^ storage reagent, and kept at -20°C following the procedure suggested by the supplier (Sigma-Aldrich, Germany). Embryos from each replicate were homogenized and RNA was then extracted using the NucleoSpin^®^ RNA Kit (Macherey-Nagel, Germany) following the manufacturer’s instructions. RNA integrity was assessed by agarose gel-electrophoresis. RNA concentration and purity were determined by spectrophotometry using Nanodrop™ One (Thermo Scientific) and normalized to a common concentration of 94 ng/μL with HPLC water. From the resulting total RNA, 850 ng were transcribed using the qScript^TM^ cDNA Synthesis Kit (Quantabio, Germany) according to the manufacturer’s instructions, including an additional gDNA wipe out step prior to transcription [PerfeCTa DNase I Kit (Quantabio, Germany)].

Quantitative real-time PCR (qRT-PCR) was used to determine the expression levels of five reference and 11 target genes (Table 2). The *ef1α*, *rps18*, *cox-1*, *atp6*, and *slc9a3*.*1* genes were chosen as housekeeping genes after calculating the geNorm stability (M-value) using the qBase+ software [56]. The analysis revealed that mRNA levels of these genes were stable throughout the analysed samples (gNorm-V < 0.15). We targeted genes related to molecular mechanisms involved in the response to acidification in fish early life stages: heat shock protein 70 (*hsp70*) and heat shock protein 90 (*hsp90*), involved in physiological stress/repair response [16, 57, 58]; aquaporin 1 and aquaporin 3 (*aqp1*, *aqp3*), which are water and solute transporters [59]; corticotropin-releasing factor receptor 1 and 2 (*crhr1*, *crfr2*), that mediate responses to acute stress [60]; Na^+^K^+^2Cl^-^ cotransporter 1α (*nkcc1a*), Na^+^Cl^-^ Cotransporter b (*ncc*), and carbonic anhydrase 15 (*car15*), involved in acid-base regulation [61, 62]; and gamma-aminobutyric acid A receptor subunit α 6b and α 1 (*GABAAα6b*, *Gabra1*), related to inhibitory neurotransmission [13]. Primers were designed using primer 3 software version 0.4.0 (http://frodo.wi.mit.edu/primer3/) based on cDNA sequences available in NCBI (https://www.ncbi.nlm.nih.gov/), preferring, when possible, the sequences associated with the latest release of the European eel reference genome (GCF_013347855.1). All primers were designed for an amplification size ranging from 75 to 200 nucleotides and optimal Tm of ~60°C and secondary structures ~0 (within and between each primer pairs).

**Table 2:** Sequences of European eel, *Anguilla anguilla* primers used for amplification of genes by qRT-PCR.

Full name	Abbreviation	Function	Accession number	Primer sequence (5’ 3’) (F: Forward; R: Reverse)
18s ribosomal RNA	*rps18*	Reference	XM_035428274.1	F: CGAGGTTGAGAGAGTGGTG R: TCAGCCTCTCCAGATCCTCT
Elongation factor 1	*ef1*	Reference	XM_035428799.1	F: CTGAAGCCTGGTATGGTGGT R: CATGGTGCATTTCCACAGAC
Cytochrome-C-Oxidase	*cox-1*	Reference	KX870839.1	F: CTACTCCTCTCCCTGCCAGT R: CTTCTGGGTGGCCGAAGAAT
ATP synthase F0 sub-unit 6	*atp6*	Reference	NC_006531.1	F: GGCCTGCTCCCATACACATT R: GACTGGTGTTCCTTCTGGCA
solute carrier family 9 member A3, tandem duplicate 1	*slc9a3*.*1*	Reference	XM_035429694.1	F: GGCGTCACCCACAATAAGATG R: GTTACAGTCATGGGCGTTCC
Aquaporin 1	*aqp1*	Water transport	XM_035431380.1	F: GAATTCCTGGCAACCTTTCA R: CAAGATGACCCAGACCCACT
Aquaporin 3	*aqp3*	Water transport	AJ_319533	F: GCTCTCATGGCTTGTTCCTC R: AAGGTCACAGTGGGGTTCAG
Heat shock protein 70	*hsp70*	Stress/repair response	AZBK_01685255	F: TCAACCCAGATGAAGCAGTG R: GCAGCAGATCCTGAACATTG
Heat shock protein 90	*hsp90*	Stress/repair response	AZBK_01838994	F: ACCATTGCCAAGTCAGGAAC R: ACTGCTCATCGTCATTGTGC
Corticotropin-releasing factor receptor 1 / corticotropin-releasing hormone receptor 1	*crhr1*	Stress response	XM_035398323.1	F: CGCTACAACACCACGAACAA R: GCGCATGAAGAGGAGGAATG
Corticotropin-releasing factor receptor 2	*crfr2*	Stress response	XM_035431405.1	F: CTGGAACCTGATCACCACCT R: GCAGTGTGAAGATAGCAGCC
Na^+^K^+^2Cl^-^Cotransporter 1α	*nkcc1a*	Ion transport	AJ486858	F: CCAAGGCTCAGATCTTCCTG R: TTTCCGAATGGTAACCGAAG
Na^+^Cl^-^Cotransporter b	*ncc*	Ion transport	AJ564606.1	F: CCTGGGAATTTCAGCTACCA R: CCTTCACACACTGCAGAGGA
Carbonic anhydrase 15	*car15*	Acid-base regulation	XM_035390336.1	F: CGGCGTTCATTGCAAATTCA R: GACATGTTCCGGCTCATCTG
GABA A receptor subunit alpha 6b	*GABAAα6b*	Neural activity regulation	XM_035433766.1	F: CATGACCACTCCCAACAAGC R: GACTCCTCTGGCACCTCTAC
Gamma-aminobutyric acid type A receptor subunit alpha1	*gabra1*	Neural activity regulation	XM_035431680.1	F: AACAATGTTGGGACGAACGG R: AGCAAGCTGTCCAGGATTCT

Expression of genes in each replicate for the samples taken at 24 and 48 hpf were analysed in three technical replicates of each gene using the qPCR Biomark^TM^ HD technology (Fluidigm) based on 48.48 dynamic arrays (GE chips). In brief, a pre-amplification step was performed with a 500 nM primer pool of all primers in TaqMan-PreAmp Master Mix (Applied Biosystems) and 1.3 μL cDNA per sample for 10 min at 95°C and then 14 cycles of 15 s at 95°C and 4 min at 60°C. Obtained PCR products were diluted 1:10 with low EDTA-TE buffer. The pre-amplified product was loaded onto the chip with Ssofast-EvaGreen Supermix low Rox (Bio Rad) and DNA-Binding Dye Sample Loading Reagent (Fluidigm). Primers were loaded onto the chip at a concentration of 50 μM. The chip was run according to the Fluidigm 48.48 PCR protocol with a Tm of 60°C. The relative quantity of target gene transcripts was normalized and measured using the 2^-ΔΔCt^ method [63]. Coefficient of variation (CV) of technical replicates was calculated and checked to be <0.04.

### 2.6. Statistical analyses

All data were analysed using SAS statistical software (version 9.1; SAS Institute Inc., Cary, North Carolina). Residuals were tested for normality using the Shapiro-Wilk test and homogeneity of variances was tested using a plot of residuals vs. fit values (PROC GLOT, SAS Institute 2003). Tukey’s post-hoc analyses were used to compare least-squares means between treatments.

The effect of pCO_2_/pH on the percentage of irregular cleavages at 4 hpf as well as egg chorion diameter and perivitelline space area at 24 hpf were determined using a series of one-way ANOVA models, where parental cross was considered a random factor (SAS PROC MIXED; SAS Institute 2003). Furthermore, mixed-model ANOVAs were used to investigate pCO_2_/pH effects on survival and gene expression throughout embryonic development. Models contained the pCO_2_/pH and age (hpf) main effects as well as the pCO_2_/pH treatment × age interaction. Akaike’s (AIC) information criterion was used to assess which covariance structure (compound symmetry, autoregressive order, or unstructured) was most appropriate [64]. pCO_2_/pH treatment and age were considered fixed, whereas parental cross was considered random. If a significant pCO_2_/pH × age interaction was detected, the model was decomposed into a series of reduced ANOVA models to determine the effect of pCO_2_/pH for each age. This was only the case for survival.

## 3. Results

### 3.1. Embryonic survival and morphological traits

**Fig 3:**
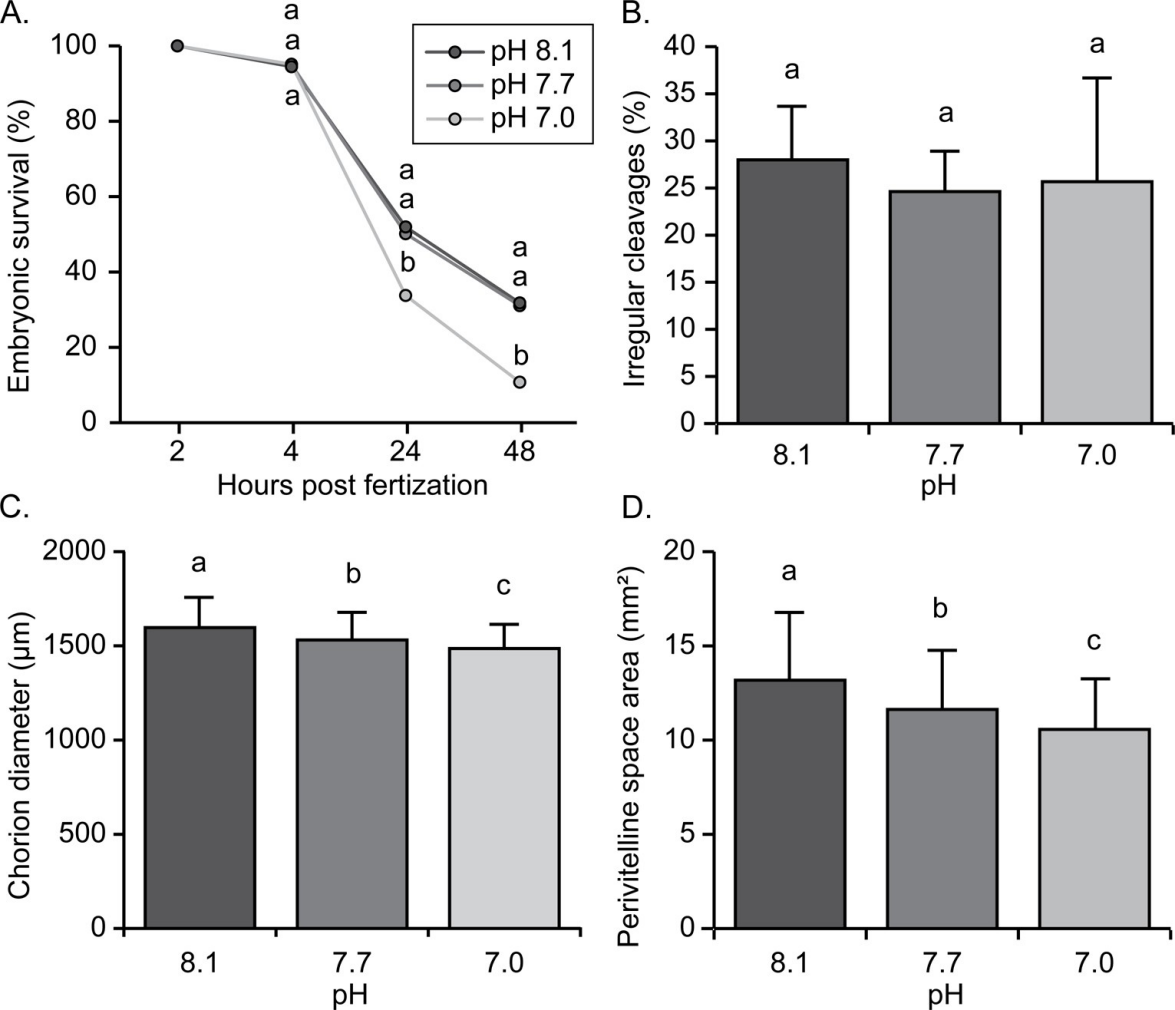
Effect of pCO_2_/pH on European eel, *Anguilla anguilla* (A) survival throughout embryonic development, (B) percentage of irregular cleavages at 4 hours post-fertilization (hpf), (C) chorion diameter at 24 hpf, and (D) area of the perivitelline space at 24 hpf. Values represent means (± SD) among 3 parental replicate crosses. Different letters represent significant differences (p < 0.05) among pCO_2_/pH treatments at each developmental stage.

There was a significant pCO_2_/pH × age interaction for embryonic survival (p = 0.0098). All three pCO_2_/pH treatments were similar at 4 hpf (p = 0.618). By 24 hpf, embryonic survival had a 0.6-fold decline under extreme acidification compared to the experimental control (p = 0.034). A similar response to pCO_2_/pH was again observed at 48 hpf (p < 0.0001), with a 0.3-fold decline compared to the control (Fig 3A).

The percentage of irregular cleavages (evaluated at 4 hpf) was similar for all pCO_2_/pH treatments (p = 0.739), with mean values ranging from 24.62 (intermediate-pH) to 27.99% (control-pH) (Fig 3B). Embryonic morphology at 24 hpf was negatively affected by decreasing pH. Specifically, egg diameter (p < 0.0001) and the size of the perivitelline space (p < 0.0001) were statistically impacted by pCO_2_/pH (Fig 3C and 3D), where both traits declined in size with decreasing pH of the rearing water.

### 3.2. Gene expression

#### 3.2.1. Stress/repair

**Fig 4:**
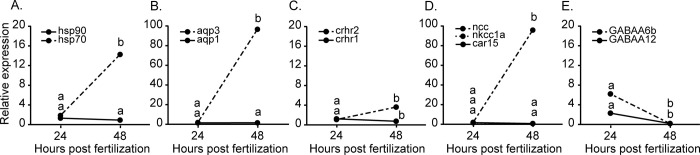
mRNA transcript abundance in European eel, *Anguilla anguilla* embryos at 24 and 48 hours post-fertilization for (A) stress/repair responses (*hsp70* and *hsp90*), (B) water and solute transport *(aqp1* and *aqp3*), (C) acute stress responses (*crhr1* and *crfr2*), (D) acid-base regulation (*car15*, *ncc*, and *nkcc1a*), and (E) inhibitory neurotransmission (*GABAAα6b* and *Gabra1*). Values represent means (± SD) among three parental replicated crosses and three pCO_2_/pH treatments at each developmental stage. Different letters represent significant differences (p < 0.05).

Expression levels of *hsp90* were similar across the pCO_2_/pH treatments and developmental times, whereas *hsp70* expression increased from 24 to 48 hpf (p < 0.0001) (Fig 4A).

#### 3.2.2. Water and solute transport

Expression levels of *aqp3* significantly increased from 24 to 48 hpf (p < 0.0001), although these levels were not affected by the pCO_2_/pH treatments. On the other hand, *aqp1* expression was similar across the pCO_2_/pH treatments and developmental times (Fig 4B).

#### 3.2.3. Stress

No significant differences were detected for *crhr1* and *crfr2* among the pCO_2_/pH treatments. However, these genes showed differences in expression patterns during development, where *crhr1* showed a decrease in expression from 24 to 48 hpf (p = 0.008), while *crfr2* expression significantly increased from 24 to 48 hpf (p = 0.0005) (Fig 4C).

#### 3.2.4. Acid-base regulation

Expression levels of *car 15* and *nkcc1a* were similar across treatments and developmental times, while *ncc* expression increased with developmental time, irrespectively of the pCO_2_/pH treatment (p < 0.0001) (Fig 4D).

#### 3.2.5. Inhibitory neurotransmission

For all pCO_2_/pH treatments, *GABAAα6b* and *Gabra1* expression decreased over time (p = 0.003; p = 0.003), although it was not significantly affected by changes in pCO_2_/pH (Fig 4E).

## 4. Discussion

Exposure to extreme acidification during European eel embryonic development had a negative impact on survival, while embryos developing at intermediate acidification showed similar survival rates to the control. Even though studies are still limited to a low number of taxa, embryonic sensitivity to acidification appears to be highly variable across fish species. For example, in the two-spotted goby, *Gobiusculus flavescens*, exposure to pH 7.6 and 1400 μatm resulted in a two-fold increase in embryonic mortality compared to current day values [65]. However, many species, such as the marine medaka, *Oryzias melastigma* [66, 67] and the Atlantic herring, *Clupea harengus* [68], are able to tolerate pCO_2_ levels ranging from 1000 to 4000 μatm (~pH 7.7–7.0) in terms of embryonic survival. Differences in life-history traits may contribute to the observed differential tolerance to acidification across fish species, as *inter alia*, the duration of the incubation period defines the time of exposure to low pH during these early vulnerable stages [69]. It has also been proposed that species that reproduce in relatively stable pCO_2_ conditions, such as in open ocean environments, would be most sensitive to high CO_2_ conditions [70, 71]. On the contrary, those species spawning and developing in coastal areas, where they encounter diel and seasonal fluctuations, ranging 200–5000 μatm, would experience a strong selection for CO_2_ tolerant phenotypes and therefore, show a decreased sensitivity towards acidification [70, 72]. Eel embryos, in particular, develop in a relatively stable open-ocean habitat, experiencing a seasonal variability of 80–100 μatm pCO_2_ in the Sargasso Sea [73]. The high embryonic mortality observed in the extreme pH treatment, is thus in agreement with the predictions made under the Ocean Variability Hypothesis [70].

Ocean acidification takes place together with global warming, pollution, and other factors that can potentially affect fish physiology. As such, sensitivity to CO_2_ is expected to increase when individuals are exposed to adverse drivers simultaneously [74]. In the current study, intermediate acidification alone, affected embryonic morphology (decreased chorion diameter and perivitelline space), but did not have a detrimental effect on embryonic survival in European eel. However, as it was reported for other fish species, when combined with other stressors, such as temperature [5, 18, 75] or hypoxia [76, 77], it could synergistically lead to an increased mortality. Such scenarios should also be considered in relation to European eel culture. Here, decreasing dissolved oxygen levels and pH during offspring culture can occur as a result of high stocking densities [78] or microbial activity [79].

At decreasing pH levels, egg swelling was impaired resulting in a reduction in egg size. Under normal conditions, this process starts with water and ion influx through the chorion from the surrounding media into the perivitelline space, following cortical alveolar exocytosis, which forms colloidal pressure, resulting in egg swelling [80]. As high concentrations of monovalent hydrogen ions have an inhibitory effect on colloidal processes, the exposure of embryos to low pH water during perivitelline space formation leads to a reduction in water uptake [81]. This consequently results in decreased egg size, as was observed for several species [82, 83]. Although egg swelling is mainly expected to occur during a short period of 0–3½ h after activation [49], alterations in water pH impedes embryonic development and may have detrimental effects [84]. In European eel embryos, water uptake continues until the 16-cell stage, when the perivitelline space reaches its maximum size [35]. In the present study, fertilized eggs were transferred to the experimental conditions after being incubated for 2 hpf at “control” conditions. Even at that stage, when they had completed the first cell cleavage and egg swelling was clearly observed, the change from control conditions to decreasing pH levels led to a reduction in perivitelline space size. Such a decrease, even though not leading to direct increased mortality, as was observed for the extreme acidification conditions, could negatively affect the embryos osmoregulatory abilities [85] and potentially lead to abnormalities during later development, which was not evaluated in this study.

Stress factors, such as increases in temperature or changes in salinity, are expected to lead to upregulation of *hsp* expression [58]. These are molecular chaperones responsible for folding and importing proteins into cellular compartments, preventing protein aggregation, refolding misfolded protein, and degrading unstable proteins [57]. In particular, heat shock response to elevated pCO_2_ in juvenile stages or adults appears to be restricted to specific tissues, directly exposed to it, such as the gills [16], or have an antagonistic effect with other stressors [42, 86]. However, it is still unclear how acidification affects *hsp* regulation during embryonic development. In the present study, *hsp70* and *hsp90* expression in European eel embryos was unaffected by changes in pCO_2_, even though these genes have been shown to be sensitive in response to other biophysical parameters such as salinity and temperature during early larval stages [33, 37]. One possible explanation would be in relation to the developmental stage at which *hsp* expression was assessed. In zebrafish, the *hsp* mechanism has constitutively expressed genes that are induced during environmental stress, providing protection to the embryo. Their regulation is stress specific and varies during development, where early embryonic stages are considered most susceptible [87]. In fact, previous studies in European eel have shown that the expression of *hsp90* peaks at 32 hpf and declines towards hatch, whereas *hsp70* expression starts to gradually increase from 32 hpf onwards [46]. In the present study, we observed a high mortality from 4 to 24 hpf, when *hsp* expression was low, and therefore, a low resistance to stressors like acidification could be expected. Moreover, *hsp* expression was evaluated at a late stage of gastrulation and during early segmentation, targeting embryos that possibly exhibited a higher heat shock response at earlier stages (*i*.*e*. before the first sampling point), which compensated potential physiological trauma and therefore were able to tolerate and survive high pCO_2_ conditions.

Aquaporins are a family of intrinsic membrane proteins that facilitate water transport across biological membranes [88, 89]. It includes water-selective aquaporins (such as *aqp1*), but also aquaglyceroporin (such as *aqp3*), which are additionally permeable to glycerol and urea [88, 90]. Furthermore, *aqp1* is thought to be involved in angiogenesis and somitogenesis [91] and possibly functions as a gas channel, transporting both, CO_2_ and NH_3_ [12, 92]. In zebrafish and killifish, *Fundulus heteroclitus*, maternally derived *aqp1* transcripts are detected during early embryonic development and during somitogenesis, whereas *aqp3* transcription increases during mid-blastula transition and gastrulation [91, 93]. Expression of *aqp1* decreases after hatch in European eel [33, 34] suggesting that it could also be maternally inherited in this species. On the other hand, *aqp3* expression remains constant from hatch until first feeding [33, 34] and is strongly inhibited by reductions in extracellular pH as was observed by heterologous expression in *Xenopus* oocytes [94]. Thus, the results from the present study show that irrespective of pCO_2_ conditions, *aqp1* expression remained constant for both embryonic stages, whereas *aqp3* expression increased during gastrulation, from 24 to 48 hpf. An inverse pattern was observed for killifish, where *aqp3* expression was first detected during the gastrula stage, peaking throughout epiboly and declining at the end of gastrulation, suggesting a possible role in water or solute transport during epiboly of the enveloping layer [91]. In this regard, besides its importance for normal physiological function in European eel early larval stages, *aqp3* might also play a role during early organogenesis and somite development.

The corticotropin-releasing factor is a neuropeptide that plays a major role in the response to stress [95, 96]. It stimulates the release of corticotropin from the pituitary, which in turn stimulates the production and release of cortisol by the kidney interrenal cells [97, 98]. There are two corticotropin-releasing factor receptors, *crhr1* and *crfr2*, that exhibit different expression patterns among tissues and along development [96, 97]. Maternally derived *crfr* transcripts are detected in zebrafish embryos, while endogenous transcription starts during early development, although it subsequently declines to undetectable levels [97]. This could explain why zebrafish, as well as several other fish species, do not start to synthesise cortisol until after hatching [98, 99]. In the present study, expression of *crhr1* in European eel embryos decreased in all treatments from 24 to 48 hpf, whereas *crfr2* showed an opposite trend. This is similar to what was observed for zebrafish embryos, where *crhr1* expression started to decline during mid-gastrulation, but *crfr2* continued to be detected until advanced stages into segmentation [97]. However, transcription levels of *crhr1* and *crfr2* were not affected by pCO_2_ conditions in our study. These results indicate that the hypothalamic-pituitary-interrenal axis might not display a functional response to stress during embryonic development in European eel and/or that the here targeted *crfr* genes might not be the best biomarkers to study stress activation in eel embryos.

Similarly, acidification is expected to alter the expression of acid-base regulatory genes. The movement of acid-base equivalents is linked with Na^+^ uptake and involves several ion transporters located in the gill ionocytes, which might take part in restoring acid-base balance during elevated environmental CO_2_ [11, 100]. We therefore examined the expression of three genes (*car15*, *nkcc1a*, and *ncc*) that are involved in ion uptake and acid-base regulation. From those, *nkcc1* is a cotransporter that mediates the entry of Na^+^, K^+^ and Cl^−^ in fish gills and is located on the basolateral side of the mitochondrion-rich cells [61, 101]. An increase in its expression was observed for European sea bass (*Dicentrarchus labrax*) juveniles when exposed to acidification in combination with changes in salinity [102]. On the other hand, downregulation of cytoplasmic carbonic anhydrase, which catalyses the hydration of CO_2_ and provides H^+^ and HCO_3_^−^ for exchange with the environment, was observed in response to elevated pCO_2_ in the gulf toadfish, *Opsanus beta* [103, 104]. The mRNA of the membrane bound isoform *car15* was also downregulated in response to acidification in embryos and larvae of the marine medaka [19]. In the present study, we found that the expression of *nkcc1* and *car15* in European eel embryos remained constantly low for all pCO_2_ levels and across developmental stages, probably indicating that the underlying regulating systems were still undeveloped and embryos might have been limited in their ability to regulate a molecular response to acidification. Conversely, *ncc* expression showed a 50-fold increase from 24 to 48 hpf for all treatments. These Na^+^Cl^-^ cotransporters are highly expressed in the yolk sac membrane of Mozambique tilapia, *Oreochromis mossambicus* [105] and in the skin mitochondrion-rich cells of zebrafish embryos [106], which is believed to be the main site for osmoregulation in fish embryos and larvae, as they do not yet have functional gills [107]. In agreement with this, the increase in *ncc* expression we observed, suggests that *ncc* may play an important role in osmoregulation already at the maternal-to-zygotic transition, with increasing functionality during embryonic development, towards early larval stages. On the other hand, *ncc* expression was not affected by reductions in seawater pH, indicating that it might not be involved in the response to acidification at the evaluated developmental stages.

When exposed to elevated pCO_2_, fish experience changes associated to acid-base compensation that could result in physiological alterations [61]. In particular, HCO_3_^−^ is accumulated in blood plasma with an associated fall in Cl^−^ concentration, which can alter normal ionic flux through gamma-aminobutyric acid type A receptors [108]. Under normal physiological conditions, these ligand-gated chloride ion channels are activated by the neurotransmitter gamma-aminobutyric acid (GABA), leading to hyperpolarization and inhibition of neurons [109]. However, as part of the compensatory mechanism aims to restore acid-base balance, HCO_3_^−^ and Cl^−^ gradients across the membranes are altered leading to a depolarization of the neuron. This reversal of the GABA A receptor has been linked to changes in behaviour and sensory responses in fish [110], while it was observed that the expression of some of the α subunits was upregulated in response to increases in pCO_2_ [14, 15]. Furthermore, GABA A receptor inhibition and overstimulation of neurons, such as it is expected to occur in relation to acid-base regulation, during embryonic development may cause adverse effects on the developing nervous system. In this regard, exposure of zebrafish embryos to fipronil (an insecticide that inhibits GABA A receptors) and abamectin (also believed to target GABA A receptors), lead to a decrease in spontaneous tail contractions, which is normally the first sign of motor activity [111, 112]. In the present study, we evaluated the expression of two GABA A receptor α subunits, *GABAAα6b* and *Gabra1*. Here, changes in their mRNA levels might reflect compensatory mechanisms to acidification [15] that, in turn, could potentially lead to decreased locomotion activity in embryos and other behavioural alterations in later developmental stages. On the other hand, the expression of both genes was unaffected by changes in water pCO_2_ and it decreased in all treatments from mid-gastrula to early-segmentation stage, which indicates that the mRNA detected could probably be maternally inherited.

Summarizing, this study provides first insights into the morphological and associated molecular changes related to seawater acidification during European eel embryonic development. In regards of ongoing climate change, we conclude that ocean acidification can potentially impair normal development in this species, as we observed that at end-of-century predicted pCO_2_ levels (intermediate-pH), water uptake and egg swelling was reduced, which translated in eggs with a smaller chorion diameter and perivitelline space. This could have a negative impact on osmoregulation during late embryonic developmental stages, egg buoyancy and potentially on hatching success. In fact, even though only buoyant embryos were sampled (from the floating layer) for all analyses, around 24 hpf and onwards we observed that some embryos started losing buoyancy and were distributed throughout the water column of the incubators in the intermediate and extreme-pH treatments. From an aquaculture perspective, we conclude that pH/CO_2_ fluctuations can have detrimental effects on offspring production, as increasing acidification caused increased mortality, especially under extreme pH conditions. However, these observed effects of acidification were not reflected by changes in gene expression of the underlying mechanisms investigated in this study, which seem to be under development during early embryonic ontogeny and thus we speculate that they are not yet matured to regulate a molecular response to acidification. Overall, we encourage further studies to address the effects of long-term exposure to acidification, beyond the embryonic stages and also in combination with other stressors that could potentially co-occur in nature, in relation to ocean acidification as well as under hatchery conditions.

## Supporting information

S1 Data (XLSX)
